# Movement behavior policies in the early childhood education and care setting: An international scoping review

**DOI:** 10.3389/fpubh.2023.1077977

**Published:** 2023-04-11

**Authors:** Elizabeth J. Wenden, Rosa Virgara, Natasha Pearce, Charley Budgeon, Hayley E. Christian

**Affiliations:** ^1^Telethon Kids Institute, The University of Western Australia, Perth, WA, Australia; ^2^School of Population and Global Health, The University of Western Australia, Perth, WA, Australia; ^3^Allied Health and Human Performance, University of South Australia, Adelaide, SA, Australia

**Keywords:** childcare, movement behavior, policy, implementation, physical activity, sedentary behavior, sleep, review

## Abstract

**Background:**

Meeting 24-h movement behavior guidelines for the early years is associated with better health and development outcomes in young children. Early childhood education and care (ECEC) is a key intervention setting however little is known about the content and implementation of movement behavior polices in this context. To inform policy development this international scoping review examined the prevalence, content, development and implementation of ECEC-specific movement behavior policies.

**Methods:**

A systematic literature search of published and gray literature since 2010 was conducted. Academic databases (*EMBASE, Cinahl, Web of Science, Proquest, Scopus, EBSCO, PubMed*) were searched. A *Google* search was undertaken and limited to the first 200 results. The Comprehensive Analysis of Policy on Physical Activity framework informed data charting.

**Results:**

Forty-three ECEC policy documents met inclusion criteria. Most policies originated in the United States, were subnational and developed with government, non-government organizations and ECEC end-users. Physical activity was specified in 59% (30–180 min/day), sedentary time in 51% (15–60 min/day) and sleep in 20% (30–120 min/day) of policies. Daily outdoor physical activity was recommended (30–160 min/day) in most policies. No policy permitted screen time for children <2 years, with 20–120 min/day for children >2 years. Most policies (80%) had accompanying resources but few provided evaluation tools (e.g., checklists; action plan templates). Many policies had not been reviewed since the publication of 24-h movement guidelines.

**Conclusion:**

Movement behavior policies in the ECEC setting are often vaguely worded, missing a comprehensive evidence base, siloed in development and often not tailored for the ‘real world.’ A focus on evidence informed ECEC-specific movement behavior policies proportionally aligned with national/international 24-h Movement Behaviors Guidelines for the Early Years is needed.

## 1. Introduction

Increasing sedentary leisure time, rising obesity rates and decreasing physical activity levels are a health concern globally including in young children (0–5 years) (1). A lack of adequate physical activity in children can lead to increased risk of high blood pressure, insulin resistance, musculo-skeletal problems, fatty liver disease and reflux (2) as well as obesity, teasing, bullying and being socially isolated. (3) Despite young children often being perceived as ‘active’, evidence shows less than 25% of young children meet 24-h movement (physical activity, sedentary behavior and sleep) guidelines (4, 5). The need to develop health-enhancing movement behaviors from an early age is important as they typically continue into adulthood (6, 7).

Guidelines on young children’s 24-h movement behaviors were recently released (8, 9). Led by Canada (9) and Australia (10) in 2017, the World Health Organization (WHO) (11), New Zealand, (12), South Africa (13), and the United Kingdom (14) also adopted evidence-based guidelines for the early years. Although similar, the small between country variation seen in the guidelines is due to the different ages at which children attend ECEC and start full-time school. The WHO 24-h movement guidelines recommend young children (0 < 5 years) accumulate: (1) at least 180 min of total physical activity per day including at least 60 min of moderate to vigorous physical activity for 3–4-year-olds, (2) no more than 60 min of sedentary screen time per day, and (3) 10–13 h of sleep per day (9–14). For infants, the recommendations are; (1) being physically active every day including at least 30 min of tummy time, (2) maximum of 60 min sedentary time and no screen time, and (3) 14–17 h of sleep per day (9–14).

Early childhood education and care (ECEC) services are well placed to influence young children’s movement behaviors (5, 8, 15). Young children can spend between 50% (0–2 years) to 90% (3–5 years) of their time in ECEC (16, 17) yet multiple studies have reported low levels of physical activity and high levels of sedentary time (18, 19). As children’s physical activity is generally accumulated between the hours of 9 am to 5 pm, (20) there is significant opportunity for movement behavior related interventions in the ECEC setting (8, 17, 20). Studies have shown that physical activity interventions in ECEC can increase young children’s physical activity levels *via* the development of staff knowledge and capacity, (2, 21) staff participation, role-modeling and promotion of physical activity (8, 22), time spent outdoors (8, 23), having large, open play spaces (22, 23), availability of portable play equipment (21, 23), and *via* the adoption of an ECEC-specific physical activity policy (8).

Early childhood education and care policies need to provide specific recommendations on the amount and type of physical activity children should do in care (7, 8). The 2001 US Institute of Medicine ECEC-specific physical activity guidelines provide an example recommending young children accumulate 15 min of physical activity per hour at ECEC and be sedentary for no more than 30 min at a time (24). This is in line with the WHO’s statement that physical activity guidelines and recommendations should measure duration, frequency, intensity and types of physical activity (25). Moreover the WHO’s recent *Global action plan on physical activity 2018–2030: More active people for a healthier world* (1) highlights the need for multi-level policies that target physical activity of sufficient duration and intensity to provide health benefits, reduce gender, social and geographic disparity and that allow tracking of progress (1). Even slight increases in physical activity can be beneficial (26) with measurement and monitoring of progress necessary to provide data about the implementation of policy actions (1). Therefore, movement policies that state specific types and amounts of movement behaviors instead of using subjective and inconsistent terms (e.g., large, small) is important for identifying progress toward 24-h movement guidelines and increasing physical activity in the ECEC setting (24).

It is currently unknown how well movement policies have been implemented into the ECEC sector (17). It is timely, therefore, to undertake an international scoping review of ECEC-specific movement behavior policies to identify where such policies exist, how they have been developed, the recommendations contained therein and the supports available to aid in their implementation.

## 2. Methods

This scoping review was undertaken using the framework developed by Arksey and O’Malley (27). The Comprehensive Analysis of Policy on Physical Activity (CAPPA) (2018) (28) framework was used to inform data collection and content analysis for the review.

### 2.1. The Comprehensive Analysis of Policy on Physical Activity framework

The CAPPA framework combines a rigorously developed methodology with flexibility to facilitate a holistic approach to data collection and knowledge synthesis for assessing physical activity policies and enables side-by-side comparison of movement behavior-related policies (28). Overall, the CAPPA framework comprises six categories (purpose of analysis, policy level, policy sector, type of policy, stage of policy cycle, scope of analysis) with 38 elements designed to examine the development, implementation, external influences, actors and stakeholders, guiding values and content of physical activity policies (28). The CAPPA framework has also been used to assess sedentary behaviors (28). The CAPPA framework definition of ‘*physical activity policy*’ (i.e., *formal written policies, unwritten formal statements, written standards and guidelines, formal procedures and informal policies*)’ (28) was deemed broad enough to capture the potentially wide array of policy documents related to movement behaviors (physical activity, sedentary behavior and sleep) in ECEC settings.

This scoping review was completed in accordance with PRISMA-ScR (29) reporting guidelines (2020) (Supplementary Appendix A). No ethics approval was required.

### 2.2. Research question

This research was guided by the following questions: (1) What is the prevalence, content and development of movement behavior policies, regulations and standards for children 0–5 years of age attending ECEC, internationally, and (2) What are the gaps, limitations and future research priorities in this area.

### 2.3. Search strategy

A set of keywords were developed using the *Web of Science* citation search tool to verify and tailor keyword selections and combinations (Supplementary Appendix B) between July – October 2021. Using these keywords, academic databases (*EMBASE, Cinahl, Web of Science, Proquest, Scopus, EBSCO, PubMed*) were comprehensively searched. A *Google* search was also undertaken using the same keyword selections and limited to the first 200 results. Reference lists were checked for additional sources of literature.

### 2.4. Study selection

All types of literature and study designs were eligible for inclusion including but not limited to; journal articles, original research including observational studies, prospective and retrospective cohort studies, systematic reviews, qualitative studies, theses, government (either state or national) reports, non-government organization (NGO) or private sector documents, websites and online resources. Inclusion criteria included literature (‘policy documents’) with an ECEC-specific movement behavior policy and/or guidelines and/or recommendations, relevant for the 0–5 age group (Table 1) (30). Policy documents were required to include a time (hours or minutes) and/or frequency provision (number of times) for at least one movement behavior (physical activity, sedentary behavior or sleep). Of these, policy documents were included in the review if they were being applied at the population level (i.e., cities, states or countries), had been published since 1st January 2010 and were available in English.

**Table 1:** Scoping review inclusion and exclusion criteria.

Inclusion criteria
Population	Young children 0–6 years of age (may cover wider age groups inclusive of 0–6 years)
Intervention	Movement behaviors (physical activity and sedentary behavior and/or sleep)
Context	Early childhood education and care (ECEC) formal long day care and/or children and/or nursery school in any city-sized or larger jurisdiction
Outcome	Policy, guidelines and/or recommendation development and implementation
Exclusion criteria
	Population	Children aged over 6 years of age
Intervention	Sedentary behaviors or sleep only
Context	Informal childcare, out-of-school care, family day care
Outcome	Intervention, impact or policy, guidelines and/or recommendation adherence reports

### 2.5. Data charting

The data charting template was uploaded into Covidence™ (31). Data was charted into a CAPPA-informed template (Supplementary Appendix C) (28). Recorded items included bibliography, document type, policy level and sectors, policy cycle and processes, actors, political will, policy content and details of specific movement behavior guidelines/recommendations.

### 2.6. Summarizing and reporting the results

Using Endnote X9, (32) the literature search results were compiled and citations exported into Covidence™ systematic review software (31) for screening and extraction. Analysis was undertaken independently by title and abstract and by two authors (EW and RV). Disagreements about inclusion were identified, discussed by the reviewers and resolved by consensus. Data synthesis was completed by EW and confirmed by RV. No quality assessment of evidence was undertaken, as per scoping review methodology (29).

## 3. Results

**Figure 1:**
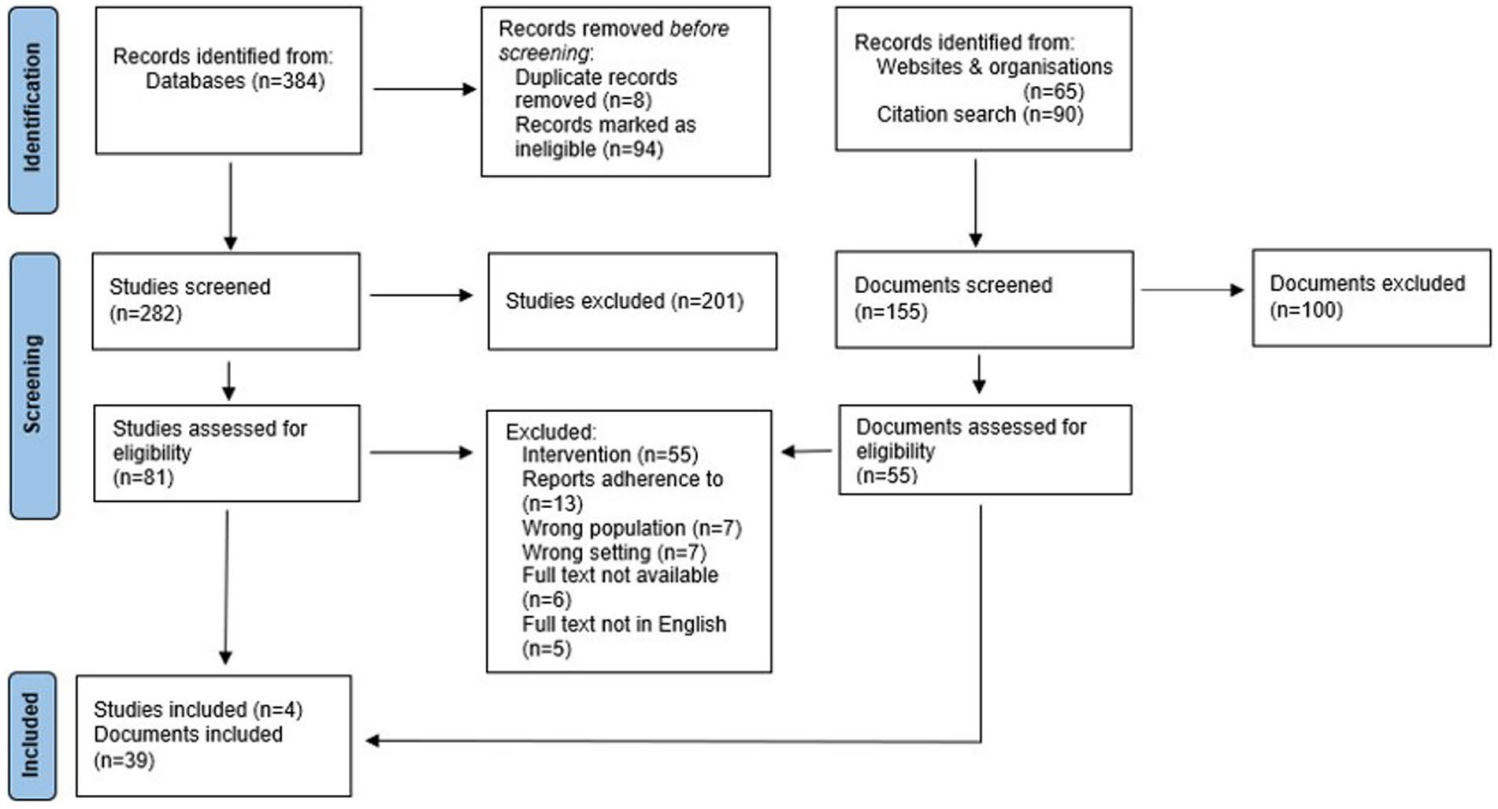
PRISMA-ScR flowchart.

The literature search resulted in 539 citations for consideration. After duplicates were removed 136 citations remained for full text review. Following full text review, 43 (4 journal articles studies and 39 other documents) policy documents were included in this review. The review process is outlined in Figure 1.

Data was synthesized into tables describing: (1) ECEC policy recommendations of duration (minutes) and/or frequency of movement behaviors (Supplementary Tables S1, S2); and (2) an overview of ECEC movement behavior policies in terms of stakeholders, development, content and supports (Supplementary Table S3). The main characteristics of included studies and documents are provided in Table 2.

**Table 2:** Summary of included ECEC movement behavior policies, guidelines and recommendations, 2010-2021.

**Title**	**Year/ update**	**Jurisdiction**	**Age group**	**Source type** ^**a**^	**Policy sectors**	**Stakeholders**	**Developmental agenda**	**Movement behavior recommendations** ^**b**^
National Database of Child Care Licensing Regulations (52)	2021	USA	0-6 years	Database (multiple US states)	Health, Education	Government	Health, safety and wellbeing practices and policies for ECEC settings	TPA, VPA, MVPA, LPA, TT, OPA, SB, ST, SL (state dependent)
Ten ways to empower children to live healthy lives (68)	2016	Arizona, USA	0-6 years	Website	Health, Education	Government, Non-government, Users	Address obesity in young children through various channels	MVPA, TT OPA, SD ST
State regulations promoting infant physical activity in early care and education (34)	2018	USA	Infants	Journal article	Health	Government, Academia, Users	Mandate ECEC physical activity requirements	TT
Child care policy and standards manual (32)	2017	Newfoundland & Labrador, Canada	0-5 years	Resource	Health, Education	Government	Mandate ECEC movement behavior requirements; supporting improved developmental outcomes for children in ECEC	TPA, OPA, ST, SL
Enacting eight policies to prevent childhood obesity: Projected outcomes for Louisiana (60)	2013	Louisiana, USA	1-5 years	Policy brief	Health, Education	Academia	Mandate ECEC physical activity requirements; Life course impact of obesity	TPA, OPA, ST
Healthy living guidelines for early learning and child care centres on Prince Edward Island (71)	2016	Prince Edward Island, Canada	0-5 years	Resource	Health, Sport, Recreation, Education	Government, Non-government, Users	Promoting health and well-being, preventing injury and shaping a caring ECEC environment	TPA, OPA, ST
Early care and education physical activity toolkit for preschool-aged children (35)	2018	California, USA	0-5 years	Resource	Health, Environment	Government, Non-government, Academia	Need for ECEC policy to increase physical activity and reduce obesity	TPA, TT, OPA, SB, ST, SL
Model child care licensing statute for obesity prevention: Sample standards for physical activity, nutrition, & screen time (65)	2013	USA	0-6 years	Statute	Health	Government, Academia	Mandate ECEC physical activity requirements; Life course impact of obesity	MVPA, OPA, SB, ST
Get up and grow: Healthy eating and physical activity guidelines for early childhood (42)	2013	Australia	0-5 years	Resource	Health	Government, Non-government, Reference group, Academia, Users	Need for ECEC policy to increase physical activity and reduce obesity	TPA, SB, ST
Director of Licensing standard of practice - Active play (70)	2016	British Columbia, Canada	0-5 years	Resource	Health, Education	Government	Mandate ECEC physical activity requirements; Address obesity in young children	TPA, MVPA, TT, OPA
Joy, play and doing together. Recommendations for physical activity in early childhood (75)	2016	Finland	0-8 years	Resource	Health, Education	Government, Non-government, Reference group, Academia, Users	Increasing the level of physical activity in ECEC through child-focused operating methods	TPA, VPA, OPA
Sit less, move more, Sleep well: Active play guidelines for under-fives (12)	2017	New Zealand	0-5 years	Resource	Health, Sport	Government, Reference groups	Increasing the level of physical activity and reducing inactivity at ECEC	TPA, ST
Play Scotland: Delivering and celebrating children and young people’s right to play - Physical activity guidelines (54)	2021	Scotland, UK	0-4 years	Website	Sport, Recreation, Environment, Urban planning	Non-government, Academia, Users	Supporting children to improve health and wellbeing and reach their full potential through child-friendly environments	TPA, MVPA, TT
State of Alaska early learning guidelines (48)	2020	Alaska, USA	0-5 years	Resource	Health, Education	Government, Non-government, Academia, Users	Mandate ECEC movement behavior requirements	TPA, VPA, TT, OPA, ST
Physical activity guide for children aged 2-6. For kindergartens and child care centres (47)	2020	Hong Kong	2-6 years	Resource	Health, Recreation, Education	Government, Non-government, Reference group, Academia, Users	Address obesity in young children in Hong Kong; Increasing the level of physical activity in ECEC	TPA, MVPA, SB, ST
Model childcare health policies (64)	2014	USA	0-6 years	Resource	Health, Education	Non-government, Academia, Users	Mandate ECEC movement behavior requirements; Reduce burden on ECEC services	MVPA, TT, OPA, SB, ST
Munch’n’move (46)	2020	New South Wales, Australia	0-5 years	Website	Health, Education	Government, Non-government, Reference group, Academia, Users	Promoting healthy movement behaviors and habits; Life course impact of obesity	TPA, VPA, TT, SB, ST
Best practices for physical activity in early care and education settings (58)	2016	North Dakota, USA	0-5 years	Resource	Health, Education	Government, Non-government, Academia	Promoting health and well-being, preventing injury and shaping a caring ECEC environment; Building educator capacity	MVPA, TT, OPA, SB, ST
Nutrition and physical activity best practices for child care centers (66)	2019	New York, USA	0-12 years	Resource	Health, Urban planning	Government, Non-government, Reference group, Academia, Users	Strengthening health, safety and wellbeing practices for ECEC settings; Building educator capacity	TPA, OPA, SB, ST
Move Smart: Physical activity best practices for child care (39)	2018	Missouri, USA	0-6 years	Resource	Health	Government	Building capacity to help services evaluate and create an environment that supports increased physical activity	TT, OPA, SB, ST
Child care licensing manual (41)	2019	Ontario, Canada	0-5 years	Resource	Education	Government, Users	Mandate ECEC movement behavior requirements; Key role of ECEC in healthy child development, well-being and learning	OPA, SL
Best practices for physical activity (57)	2013	USA	0-18 years	Resource	Health	Non-government, Reference group, Academia, Users	Need for ECEC policy to increase physical activity and reduce obesity in children	TPA< TT, OPA,SB, ST
Growing healthy children: A guide to enhance nutrition and physical activity in New York City group child care centers (61)	2011	New York, USA	0-5 years	Resource	Health	Government	Need for ECEC policy to increase physical activity and reduce obesity; Enable children to be physically active in places where they live, learn and play	TPA, OPA, SB, ST
Let’s go!: Early care and education programs (63)	2015	Maine, USA	0-5 years	Website	Health, Environment	Non-government, Reference group	Need for ECEC policy to increase physical activity and reduce obesity; Life course impact of obesity	TPA, ST
Early child care obesity prevention recommendations: Complete list (59)	2011	Massachusetts, USA	0-5 years	Website	Health, Education	Government, Non-government, Reference group, Academia, Users	ECEC educators as change agents; Key role of ECEC in healthy child development, well-being and learning	TPA, TT, OPA, SB, ST
Setting the table - nutritional guidance and food standards for early years childcare providers in Scotland (37)	2018	Scotland, UK	0-16 years	Resource	Health	Government	Reducing health inequities; Promoting healthy physical activity habits	TPA, OPA,
Healthy kids, healthy futures (55)	2021	USA	0-5 years	Website	Health	Government, Non-government	Improving health where it starts - at home, in the community and anywhere you find children, including ECEC	MVPA, TT, OPA, ST
Active early: A Wisconsin guide for improving childhood physical activity (56)	2011	Wisconsin, USA	0-5 years	Resource	Health, Education	Government, Non-government, Reference group, Academia, Users	Key role of ECEC in healthy child development, well-being and learning; promotion of healthy habits at ECEC and in the home	TPA, OPA, SB
Iowa early learning standards (36)	2018	Iowa, USA	0-5 years	Resource	Education	Government, Non-government, Reference group, Academia, Users	Mandate ECEC physical activity requirements; Promotion of healthy child development for the best life chances	TT, OPA. SB
Colorado early learning & development guidelines (44)	2010	Colorado, USA	0-5 years	Resource	Health, Education	Government, Non-government, Reference group, Academia, Users	Standardize and mandate health, safety and wellbeing practices and policies for ECEC settings	VPA, TT, OPA
NB plays! Preschool - A resource for quality early learning programming (72)	2016	New Brunswick, Canada	0-5 years	Resource	Health, Sport, Education	Government, Non-government, Users	Promoting physical activity behaviors to support holistic child development; Life course impact of healthy habits	TPA, MVPA, OPA, ST
Early movers. Helping under-5s live active & healthy lives: Introduction to physical activity in the early years (74)	2012	UK	0-5 years	Resource	Health, Education	Government, Reference group, Academia	Early introduction of sufficient physical activity encourages children to stay active throughout the life course	TPA
Policy perceptions related to physical activity and healthy eating in Mississippi (67)	2013	Mississippi, USA	0-18 years	Journal article	Education	Academia, Users	To involve researchers and policymakers in a discussion to tailor policies to impact obesity rates across the life course	TPA
The physical activity guidelines for Americans (40)	2018	USA	0+ years	Resource	Health, Sport, Recreation, Education, Transport, Environment, Urban planning	Government, Non-government, Reference group, Academia, Users	Foster normal growth and development, make people feel, function, sleep better, and reduce the risk of chronic diseases for all age groups and settings, including ECEC	TPA
Evaluation of the dissemination of the South African 24-hour movement guidelines for birth to 5 years (49)	2021	South Africa	0-5 years	Journal article	Health, Sport, Education	Government, Non-government, Reference group, Academia, Users	Movement behaviors essential for obesity prevention and developmental outcomes that are important in early childhood; Prevalence of obesity rates and NCIDs across the life course	TPA, TT, SB, ST
Development of physical activity policy and implementation strategies for early childhood education and care settings using the Delphi process (43)	2020	Perth, Australia	0-5 years	Journal article	Health, Sport, Recreation, Education	Government, Non-government, Reference group, Academia, Users	To gain consensus on evidence informed ECEC physical activity policy template; Determine best-practice dissemination and implementation strategies	TPA, TT, SB, ST
Section F: Ministerial requirements for the daily program (50)	2021	Nova Scotia, Canada	0-5 years	Resource	Education	Government	Mandate ECEC physical activity requirements	OPA
Policy on physical activity, sport and recreation (51)	2021	Quebec, Canada	0-5 years	Website	Sport, Recreation, Education	Government, Non-government	A global perspective on promoting physical activity behaviors for all populations and the different spheres of their lives, including children at ECEC	TPA
Caring for Our Children: National Health and Safety Performance Standards - Guidelines for Early Care and Education Programs (53)	2021	USA	0-6 years	Database	Health, Education	Government, Non-government, Reference group, Academia, Users	Health, safety and wellbeing practices and policies for ECEC settings	MVPA, TT, OPA, SB, ST
Institute of Medicine (IOM) Early childhood obesity prevention policies (62)	2011	USA	0-5 years	Report	Health	Government, Academia, Users	Focus attention on factors and how to mitigate overweight and obesity in infants, toddlers, and preschool children through nutrition, physical activity, and sedentary behaviors	TPA, ST
Improving healthy weight in children: The healthiest next generation initiative (76)	2014	USA	0-5 years	Resource	Health, Sport, Education, Transport	Government, Non-government- Reference group	Update health, safety and wellbeing practices and policies for ECEC settings; Build capacity in educators to meet mandated movement behaviors	VPA, TT, OPA, ST
Best practices licensing manual for early learning and childcare centres. Province of Manitoba (69)	2014	Manitoba, Canada	0-12 years	Resource	Education	Government, Non-government, Users	Standardize and update mandated health, safety and wellbeing practices and policies for ECEC settings	TT, OPA, SB, SL
Understanding Nunavut's child day care regulations: A manual for early childhood programs (73)	2014	Nunavut, Canada	0-5 years	Resource	Education	Government, Users	Health, safety and wellbeing practices and policies for ECEC settings	TPA, OPA, SL

a Source type: Database, online searchable structured dataset; Website, numerous web pages listed under one domain name; Journal article, peer-reviewed and published research, analysis or review; Policy brief, summary of issues; Legislation, enacted law; Resource, printable materials including advice and/or standards and/or policy template for physical activity.

b TPA, Total physical activity; VPA, Vigorous physical activity; MVPA, Moderate-vigorous physical activity; MPA, Moderate physical activity; LPA, Light physical activity; TT, Tummy time; OPA, Other physical activity;, SB, Sedentary behaviors SL, Sleep.

Most policy documents (74%) were published or updated in the last 5 years (12, 33, 35, 36, 39, 41, 44–46, 48, 51, 52, 57, 58, 60, 61, 65–70, 75–77) and were from high income countries. Three-quarters (75%) were published in the United States (24, 33–35, 37, 39, 40, 45, 47, 49–51, 53–56, 58–61, 64, 65, 70–72, 75), followed by Canada (12%), (36, 38, 42, 52, 62, 68, 69, 73, 74, 76) Australia (4%), (41, 48, 67) United Kingdom (4%), (44, 57, 63) and Finland, (43) South Africa, (66) Hong Kong (46), and New Zealand (12) (combined total 5%). Policy documents were located on websites (i.e., web pages listed under one domain name), in resources from websites (i.e., printable materials including advice and/or standards and/or policy template for physical activity) or online databases (i.e., online searchable structured dataset) (80%) (12, 33, 34, 36, 38, 39, 41–63, 68–70, 72–74, 76) with peer-reviewed journal articles (13%) (35, 64–67, 71, 75) and policy briefs/legislation (7%) (37, 40, 77) accounting for the remainder. While the focus was on ECEC-specific policy documents for children 0–5 years of age, over half of the policy documents (55%) included children up to the age of 6 years (12, 33–42, 44–49, 51, 52, 54–56, 58–63, 66–70, 74) with the remainder including children up to the ages of 8 or older (43, 50, 53, 57, 64, 73).

### 3.1. Movement behavior recommendations

Over half (59%) of policy documents specified an amount of total physical activity (TPA) per day for children attending ECEC (Supplementary Table S1) (12, 24, 33, 36–39, 41–45, 48, 50, 53–57, 59, 62–67, 69, 72, 74). However, there were wide variations in these policies in the amount of TPA recommended with 60 min/day most commonly recommended (43%) (33, 37, 38, 43, 48, 50, 54, 55, 69). Remaining policy documents stated 180 min/day (27%), (19, 33, 41, 43, 57, 62, 63, 66) 120 min/day (16%), (24, 33, 39, 42, 53, 67) 30 min/day (7%) (33, 45, 64, 74) and between 60 and 120 min/day (7%) (33, 36, 53, 56, 59, 67). Daily recommended minutes of moderate-to-vigorous physical activity (MPVA) were provided in 17% of all policy documents (33, 34, 40, 42, 44, 46, 47, 49, 58, 62, 70). For all but one of these, 60 min/day or more of MVPA was recommended. Daily recommended minutes of vigorous physical activity (VPA) minutes were stated in 8% of policy documents and ranged from 20–120 min/day (33, 43, 45, 48, 61, 72). Light physical activity (LPA) was specified in one policy document (33). For infants, tummy time recommendations were included in 36% of policy documents with three main types of recommendations; 30 min/day (22%), (33, 44, 48, 66, 67) at least daily (48%) (33, 35, 45, 49, 56, 60, 61, 72, 73) and 3–5 min at a time – multiple times daily (30%) (33, 34, 39, 47, 51, 53, 58, 70).

Overall, 51% of policy documents specified the duration children could be sedentary at any one time at ECEC (Supplementary Table S2) (33, 34, 39–41, 46–51, 53, 54, 56, 59, 60, 66, 67, 70, 73). There were wide variations in sedentary time duration (depending on age group) in these policies with 34% stating 60 min/day, (33, 34, 39–41, 46, 48, 53, 54, 66, 67) 34% stating 30 min/day (33, 50, 73) and 32% stating 15–30 min/day (33, 39, 40, 47, 49, 51, 53, 56, 59, 60, 70). Separate screen and sedentary recommendation were found in 61% of policy documents (33, 34, 39–41, 46–51, 53, 56, 66, 70, 78). Most policy documents did not allow for any screen time for children under 2 years of age (12, 24, 33, 34, 36–41, 47–51, 53, 54, 56, 58, 62, 66, 70, 72) while a handful allowed screen time for children over 12 or 18 months of age (33, 39, 45, 67). Where screen time was allowed the amount ranged between 15 and 120 min/day at ECEC. Several policy documents included computer time as a separate recommendation to screen time. Where computer time recommendations were provided children under 2 years of age were not permitted any while children over the age of 2 years were allocated between 15 and 120 min/day (33, 40, 41, 47, 52).

Sleep was integrated into 20% of policy documents (33, 36, 39, 52, 73, 74). Recommendations were either between 30 and 120 min/day at ECEC (40%) (33, 39, 52), between 60 and 120 min/day once the child was in care for more than 4 h (40%) (33) or specified as ‘daily’ sleep at ECEC (20%) (36, 73, 74).

### 3.2. Policy development and implementation

Overall, 50% of policy documents specifically included a statement about the need for co-operation and collaboration between government and non-government actors to support the successful implementation of ECEC movement behavior policies (Supplementary Table S3) (37, 38, 40, 41, 43, 44, 47, 48, 51, 55, 58, 60, 65, 67, 69, 72, 75, 76, 79, 80).

Thematic analysis was used to identify overarching stakeholder values in the development of policy documents:

– A recognized need to address the serious public health issue of rising obesity, increasing sedentary behaviors and decreasing physical activity in childhood (34, 37, 39, 42, 48, 53, 55, 58, 64, 66, 71).
– The desire to help children reach their full potential through supporting their health and development (36, 43, 44, 52, 59, 62, 65, 66, 69, 70, 75).
– To address health inequities in early childhood and reduce the risk of short- and long-term chronic disease (36, 46, 55–57, 62, 63, 65, 66, 69, 75, 76).
– To improve ECEC settings by building capacity to support healthy movement behaviors and facilitate supportive environments for physical activity (33, 38, 43, 44, 51, 53, 55, 59, 63, 64, 70, 72, 73, 75).
– A need to standardize and clarify the recommended amount of movement behaviors for 0–5-year-olds while attending ECEC (33, 35, 40, 41, 43, 45, 47, 50, 51, 54, 60, 61, 67, 68, 71, 73, 74, 77).

Reflecting the motivations and interest of stakeholders involved, these themes provide a summary of the values present during the early stages of the policy cycle. However, very little contextual information was provided about the conditions (e.g., economic, political, social, environmental) that led to the development of movement behavior policy documents. A closer look at the dominant values of the major stakeholders, however, provided some insight to the social, community and heath context that underpinned the policy documents. These values included: a life course perspective to promoting the health and well-being of young children, (12, 35, 37, 40, 41, 46, 50, 51, 58–61, 63, 66) the rights of children to learn, grow and develop in a safe and healthy environment, (33, 43, 44, 46, 48, 49, 56, 57, 66, 70) integration of families, educators and communities to support healthy habits in young children (34, 39, 41, 45, 48, 50, 53, 55, 56, 58, 59) and provision of physical activity in ECEC through best practice policy (47, 49, 53, 54, 64, 65, 67, 71, 72, 75).

Most policy documents (83%) were enacted at the subnational level, i.e., state or province, (34, 36–39, 42, 44, 48–52, 54–57, 59–62, 64, 67–69, 73–75, 77) with national policy documents representing the remaining 17% (12, 33, 35, 40, 41, 43, 46, 47, 53, 58, 63, 65, 66, 70–72, 76). Implementation supports such as resource/practice guides and training were provided as part of 80% of policy documents (12, 34–36, 38–68, 70, 73, 74), 57% provided dedicated websites (12, 34, 38, 39, 41, 43–46, 48, 51, 53, 55–58, 60, 62–64, 66, 67, 70, 71, 73, 74, 76), and/or other implementation tools (e.g., document templates, practice examples) (34, 36, 38–40, 43, 44, 46, 48, 50–53, 55–59, 61, 63, 66, 67, 69, 73, 74, 76) and 28% provided self-evaluation tools (e.g., checklists, periodic surveys, action plans) (34, 39, 44, 46, 48, 50, 51, 53–55, 57–59, 67).

The majority of policy documents (81%) were developed by the jurisdiction’s health sector (12, 34–43, 45–51, 53–59, 61–67, 70–72, 75, 76). Some (28%) jurisdictions (predominately in the United States) had either a mandated regulation or made state funding reliant upon ECEC services meeting specific standards or criteria including ECEC-specific physical activity policy recommendations (33, 35, 36, 42, 43, 45, 50, 52, 60, 68, 73, 74, 77). The education sector (33, 34, 36–38, 42, 43, 45–49, 52, 56, 59–63, 65–70, 72–77) and sport and recreation sectors (12, 38, 44, 46, 62, 65–67, 69, 72, 76) participated in the development of 66 and 36% of policy documents, respectively. The remaining stakeholders were from research (15%), (41, 47, 49, 51, 54, 71, 77) environment (13%), (39, 44, 55, 65, 75, 76) rural and urban planning (9%) (44, 50, 65, 76) and transport (6%) policy sectors (39, 44, 50, 55, 65, 72, 75, 76). Half of policy documents reviewed involved at least both health and education sectors (51%) while only the US *‘The Physical Activity Guidelines for Americans’* (65) and Canada’s *‘Let us get moving: A Common Vision for Increasing Physical Activity and Reducing Sedentary Living in Canada’* (55) included all major policy sectors.

The majority of policy documents were in the implementation stage (12, 34, 36, 39, 40, 42, 43, 45, 46, 50–63, 68, 69, 77) or evaluation and maintenance stages (33, 35, 38, 41, 44, 47, 48, 66, 70, 74, 75). Some documents (25%) provided evidence of agenda setting (64, 65, 72) or formulation (37, 56, 67, 71, 76) stages of the policy cycle. Policy development was generally underpinned by a range of published literature, expert opinions, as well as results from jurisdictional surveys and/or focus groups involving stakeholders from various sectors of the community.

Most policy documents were formulated in conjunction with a range of stakeholder groups although eight (17%) (33, 36, 51, 52, 54, 68, 74, 77) did not specify any stakeholders other than government departments. Further, six (13%) (37, 44, 47, 53, 55, 64) did not mention government departments at all, although some did call for government involvement and/or funding in implementing ECEC movement behavior policies. A minimum of three stakeholder groups (33, 34, 38, 39, 41, 43–50, 53, 56, 59–63, 65–67, 70–73, 75, 76) participated in the formulation in 60% of policies/guidelines/recommendations however only 30% overall cited a full range of stakeholder groups across government, non-government organizations, a working or reference group, academics and ECEC end users (41, 43, 46, 48, 50, 56, 59–61, 65–67, 70, 76). Overall, government departments were the main stakeholder named in the development of the majority (85%) of policy documents (12, 33–36, 38–43, 45, 46, 48–51, 54, 56, 58–63, 65–77).

## 4. Discussion

This scoping review identified the prevalence, content and development of ECEC movement behavior policies, regulations and standards internationally highlighting evidence gaps, limitations and future research priorities. We summarized 43 policy documents guided by the CAPPA framework (81).

There was considerable variation in the recommended amount (duration and/or frequency) of movement behaviors in ECEC policies. We also found that many policies contained recommendations for only one or two behaviors but not all three. These findings were not unexpected, given that many policies were published prior to the 2019 WHO *Guidelines on physical activity, sedentary behavior and sleep for children under 5 years of age* (11). It was a strength, however, that many policies included developmentally appropriate recommendations by age group for each movement behavior. Recommendations ranged between 60 and 180 min per day for TPA and MVPA ranged between 20 and 90 min per day. Infant tummy time recommendations were most commonly 3–5 min at a time (multiple times/day). Recommendations for the amount of structured and unstructured physical activity children should do at ECEC ranged between 30 and 60 min/day. ECEC sedentary and screen time recommendations varied with some policies including screen time as part of overall sedentary time recommendations and others specifying separate recommendations. For children over 2 years old screen time recommendations ranged between 15 and 120 min/day. Similarly, there was wide variability for ECEC sleep time recommendations with recommendations ranging between 30 and 120 min/day depending on the length of time in care.

The variation in recommended amounts of movement behaviors at ECEC reflects the limited evidence base on which these policies have been developed and most do not comprehensively reflect the WHO *Guidelines on physical activity, sedentary behavior and sleep for children under 5 years of age* (11). Small sample sizes - and consequently power issues - plague the early years ECEC movement behavior policy evidence base (4, 17, 82). Movement behavior measurement at ECEC has typically involved parent-report and proxy measures (4, 17, 82, 83). However, devices (e.g., accelerometers) capable of measuring the complete range and type of young children’s movement behaviors (including sleep) at ECEC have shown promise (84). Currently, there is debate over the use of cut points for processing accelerometer data with recent methodological studies proving machine learning algorithms to be more accurate (4, 83). In addition, few studies have outlined the processes for ECEC-specific movement behavior policy development. Given the limited amount of published literature on movement behavior policies in the ECEC setting the potential for methodological issues to influence movement behavior recommendations cannot be ignored. Therefore, more research is necessary to develop, test and implement ECEC-specific measures that expand the evidence base for movement behaviors in the ECEC setting. Attending to these issues is an important step in legitimizing the evidence base and ensuring that movement behavior recommendations are pragmatic, valid and consistent.

Overall, the vast majority of movement behavior policies were ‘downstream’, i.e., focused on the ECEC end-user, and most contained some type of support for implementing the policy. The most common policy implementation supports were resource/practice guides, professional development, templates or websites. To ensure the successful implementation of movement behavior policies in ECEC, supports need to address implementation barriers for ECEC services, be contextually specific and supported by theoretical underpinnings. It is imperative, however, that the most useful implementation supports are chosen. While the evidence shows a general insufficiency of implementation supports to influence young children’s movement behavior change (78, 85, 86) it should be acknowledged that implementation best practice in the ECEC setting is still emerging (17, 87). Further, to address barriers identified by ECEC services, implementation supports should be ongoing to ensure transfer of knowledge to new ECEC staff in a traditionally high turnover work setting (82). Specifically, supports should be tailored to overcome barriers in the ECEC setting (88) that can influence the implementation success of an ECEC-specific movement behavior policy.

The large variation in the structure of ECEC-specific movement behavior policies made it challenging to assess policy processes, content and implementation. We also noted that there was little mention of the political, social, economic or cultural contexts in which the stakeholders operated and ECEC-specific movement behavior policies were developed. Framing and development of policy is dependent on the overarching agendas of who is proposing them (89). The inherent risk is that the resulting movement behavior policy may not be sufficiently pragmatic or reflect the complexity of the ECEC setting. Furthermore, future ECEC-specific movement behavior policy development should include experts and researchers who have comprehensive knowledge of the ECEC movement behavior field to ensure the policy, its recommendations and implementation supports are backed by current evidence.

The majority of movement behavior policies originated in high-income countries. This finding was not unexpected and while ECEC-specific movement behavior policies have become more widespread internationally in recent years, they remain the purview of wealthy countries (17). With an estimated 80% of deaths attributed to non-communicable diseases occurring in low to middle income countries (90, 91). healthy physical activity habits can improve short- and long-term health outcomes and reduce the economic cost of disease (11). More research, therefore, should be conducted to understand and address the specific barriers to the development and implementation of ECEC-specific movement behavior policies in low-middle income countries.

Most movement behavior policies reviewed were subnational and from the health, education and sport/recreation policy sectors. While most collaborations for policy agenda setting and development occurred at the local level, (92) there was little intersectoral collaboration in the development and implementation of ECEC-specific movement behavior policies. Government was the main stakeholder in the development of ECEC-specific movement behavior policies, with other stakeholder groups (community, end users, academia) also well represented. This is important as stakeholders, particularly end users (ECEC providers and related peak bodies), play key roles because of their interest, overarching values and potential influence over decision making and implementation of policies in the ECEC sector (89). While acknowledging it is complex and resource intensive, (93) intersectoral collaboration supports the development of strong stakeholder relationships and ‘win-win’ strategies (92, 93) to better support the development and implementation of ECEC-specific movement behavior policies. Notably, a recent umbrella review highlighted the importance of stakeholders and political will for legitimacy in the successful implementation of physical activity policies in various populations including young children (94). This position is reflected in the WHO’s *Global Action Plan on Physical Activity 2018–2030* (1) whereby multisectoral collaboration underpins the strengthening of development, implementation and monitoring of physical activity targets. Going forward, policymakers should aim to develop broad, multi-sectoral collaborations to assist with effective ECEC-specific movement behavior policy development, implementation and outcomes.

Less than one-third of movement behavior policies provided tools, e.g., checklists, periodic surveys, action plans for individual- or service-level self-evaluation, to assess implementation progress or compliance. Yet, national regulatory authorities in many countries (e.g., Australia, United States, United Kingdom, Japan Norway, Poland) assess compliance with national ECEC movement behavior-related policies and standards (95). Overall, we found that USA movement behavior policies were legislated and often linked to service funding. In other countries, such as Australia and New Zealand, policies were not legislated but linked to ECEC quality/standards. For example, in Australia, ECEC is heavily regulated by the Australian Children’s Education & Care Quality Authority and for movement behaviors states ‘Each child’s health and physical activity is supported and promoted’ (96). Conversely, USA young children’s ECEC movement behaviors policies contain specific requirements [e.g., 60–90 min/day TPA; no screen time <2 years, 60 min/day for >2 years (33)] that are legislated. Legislated policies, such as those seen in the USA, have the potential to influence how well an ECEC-specific movement behavior policy is implemented and subsequently practiced. Further, legislation that proportionally reflects current evidence, i.e., 24-Hour Movement Guidelines for the Early Years (9–11, 13) may provide an impetus to meet policy recommendations. In conjunction with improving young children’s health and development, ECEC-specific movement behavior legislation facilitates increased policy compliance, drives educator and service capacity building, embeds policy, normalizes practice (81, 88, 97, 98) and can be leveraged off existing national quality monitoring processes. Importantly, the presence of ECEC-specific movement behavior legislation aligns with other already-legislated ECEC service standards around nutrition, sun protection and space requirements.

### 4.1. Limitations

While a scoping review can identify a broad range of evidence it does not contain a mechanism for assessing the methodological rigor or quality of that evidence. However, given the nature and stage of the field, a scoping review allowed for the mapping of the body of ECEC-specific movement behavior literature (27). Only English-language documents were included in the review and this criterion likely prevented the location of ECEC-specific ECEC movement policies in countries where English is not the primary language. We also acknowledge that while every effort was made to locate all relevant documents it is possible that some relevant literature may not have been included based on our search strategy and inclusion criteria.

## 5. Conclusion

This international scoping review identified the prevalence, content and development of ECEC-specific movement behavior policies and provided insight into the limitations, gaps and future research priorities in this area. Movement behavior policies in the ECEC setting are often vaguely worded, lacking a comprehensive evidence base, siloed in development and often not tailored for the ‘real world’. Moreover, no ECEC-specific recommendations for movement behaviors currently exist. Our findings highlighted that there are wide variations in the duration and frequency of recommendations in ECEC movement behavior policies. More research is needed to strengthen the evidence informing the amount of movement behaviors children should do at ECEC as well as the application of this evidence base in the development of ECEC-specific movement behavior policy. In particular, ECEC-specific movement behavior policies need to be proportionally aligned with national/international 24-h Movement Behavior Guidelines for the Early Years. Additionally, further work is required to develop effective and wide intersectoral collaborations of government and non-government organizations, increase stakeholder engagement and advocacy for legislated movement behavior policies. While implementation supports are provided, it is imperative they are backed by evidence and responsive to the needs of the ECEC services they were designed for. Overall, our findings indicate that evidence informed ECEC-specific movement behavior policies for the Early Years are needed to ensure young children attending ECEC have sufficient opportunity to be active.

## Data availability statement

The original contributions presented in the study are included in the article/Supplementary material, further inquiries can be directed to the corresponding author.

## Author contributions

EW: conceptualization, methodology, data search, extraction and analysis, writing – original draft, review and editing. RV: methodology, data extraction, writing – review and editing. NP: conceptualization, writing – review and editing. CB: methodology, writing – review and editing. HC: conceptualization, methodology, supervision, writing – review and editing. All authors contributed to the article and approved the submitted version.

## Funding

This work is supported by the National Health and Medical Research Council (NHMRC) partnership project grant (#APP1152086) and partially through the Australian Research Council’s Centre of Excellence for Children and Families over the Life Course (#CE200100025). EW is supported by an Australian Government Research Training Program (RTP) Stipend and RTP Fee-Offset Scholarship through The University of Western Australia and a Minderoo Foundation Top-up Scholarship through the Telethon Kids Institute. NP is supported by the Australian Government through the Australian Research Council’s Centre of Excellence for Children and Families over the Life Course (CE200100025). HC is supported by a National Heart Foundation Future Leader Fellowship (#102549). None of the funding bodies had a role in study design, data collection, analysis, report writing or publication of this article.

## Conflict of interest

The authors declare that the research was conducted in the absence of any commercial or financial relationships that could be construed as a potential conflict of interest.

## Publisher’s note

All claims expressed in this article are solely those of the authors and do not necessarily represent those of their affiliated organizations, or those of the publisher, the editors and the reviewers. Any product that may be evaluated in this article, or claim that may be made by its manufacturer, is not guaranteed or endorsed by the publisher.

## Supplementary material

The Supplementary material for this article can be found online at: https://www.frontiersin.org/articles/10.3389/fpubh.2023.1077977/full#supplementary-material
