# Supplementary material for: Movement behavior policies in the early childhood education and care setting: An international scoping review
Source: Front Public Health. 2023 Apr 11;11:1077977. doi: 10.3389/fpubh.2023.1077977 (PMC10126357; doi:10.3389/fpubh.2023.1077977)
Supplement: Supplementary file 2 [file Table_2.DOCX]

**Appendix B. Examples of scoping review search strategy.**

| **Web of Science** | |
| --- | --- |
| Terms: | ALL= (physical activity AND sedentary OR sleep OR screen*) AND ALL= (early years OR preschool OR child* OR kinder*) AND ALL= (policy OR guideline OR recommend* OR statement OR strategy) AND DOP =2010-01-01 – 2021-10-31 |
| **Google** | |
| Terms: | “Early Childhood Education and Care”; (Kindergarten or Preschool or Child*), (Physical* or “Screen time” or Sedentary or Sleep), Obesity, (Guideline or Statement or Policy or Recommendation or Strategy) |
