# Supplementary material for: Movement behavior policies in the early childhood education and care setting: An international scoping review
Source: Front Public Health. 2023 Apr 11;11:1077977. doi: 10.3389/fpubh.2023.1077977 (PMC10126357; doi:10.3389/fpubh.2023.1077977)
Supplement: Supplementary file 3 [file Table_3.DOCX]

**Appendix C. Adapted CAPPA data extraction template**

| **Document ID:** | | | **Title:** | | | | | | | |
| --- | --- | --- | --- | --- | --- | --- | --- | --- | --- | --- |
| **Country of origin:** | - US | - UK | | | | - Canada | | - Australia | |  |
|  | Other __________________________________ | | | | | | | | |  |
| **Document type:** | - Policy | - Guidelines | | | | - Recommendations | | - Protocol | |  |
|  | - Published study | - Dissemination/ Implementation | | | | - Other _______________________ | | | |  |
| **Policy level:** | - International | - National | | | - Subnational | | - Local | | - Institutional |  |
| **Policy sector:** | - Health | - Sport | | | - Education | | - Transport | | - Environment |  |
|  | - Recreation & leisure | | | - Urban/Rural planning & design | | | | | |  |
| **Policy stage** | - Formulation - What informed the formulation of the ECEC PA strategy?; Who participated in the development of the policy? | | | | | | | | |  |
|  | - Endorsement - Which bodies advocated for the adoption of the national PA strategy?; Which official body enacted the policy? How was the policy enacted, that is, did it involve legislative or executive approval or both? | | | | | | | | |  |
|  | - Implementation Was the policy implemented as intended?; How was the policy implemented?; Theoretical basis/model for implementation?; Which bodies participated in the implementation of the policy? | | | | | | | | |  |
|  | - Evaluation - What procedures are in place for evaluation of the l PA strategy?; What formal procedures are in place to determine the impact of the PA strategy? | | | | | | | | |  |
|  | - Maintenance, Termination, Succession - Who made the decision about the policy maintenance?; Were there any specific economic circumstances around the development of the ECEC PA policy?; What budget has been allocated for the implementation of the ECEC PA policy?; What are the dominant values held by the body endorsing the ECEC PA policy?; What influence does private sector have on policy making process? | | | | | | | | |  |
| **Processes:** | - What processes did the ECEC PA policy have to go through to become implemented (e.g., after Minister’s proposal, the strategy was approved by the Parliament; only one ministry approved and issued the strategy; or several ministries issued the strategy but it was not sent to the Parliament etc.)? | | | | | | | | |  |
|  | - Which mechanisms are in place to support the dissemination of PA guidelines (e.g., communication strategy)? | | | | | | | | |  |
|  | - Did the development process of the ECEC PA policy allow for suggestions and improvements to be made? | | | | | | | | |  |
| **Actors:** | - Which bodies proposed the national PA strategy? - Who were the actors involved in the development of the ECEC PA policy? - Are any non-governmental organisations assisting in the implementation of the ECEC PA policy? - What were the power relations between the actors involved in the development of the national PA strategy? | | | | | | | | |  |
| **Political will:** | - Did any political actor in power publicly express support to the development of the national PA strategy? | | | | | | | | |  |
| **Content Analysis of the wording and substantive information:** | - Does the ECEC PA policy reference specific target groups? - Does the ECEC PA policy have a clear statement on the timeframe for policy implementation? - Does the national PA strategy mention joint collaboration at different levels of government (e.g., local, regional, state)? | | | | | | | | |  |
| **Policy content:** | - Recommendations of time for any or all movement behaviours? - Guidelines for practice? - Implementation tools? - Evaluation tools? - Resource guide or similar for practitioners? - Website? - Contact for assistance? - Evidence and/or research based? - Other? _____________________________________________ | | | | | | | | |  |
| **Movement behaviours:** | - Age groups - What are the PA recommendations for the age groups in the document? - What are the SB recommendations for the age groups in the document? - What are the SCREEN TIME recommendations for the age groups in the document? - What are the SLEEP recommendations for the age groups in the document? - Is the policy content predominantly downstream (education, information) or upstream (legislation, standards, change of the environment)? | | | | | | | | |  |

ECEC, Early childhood education and care

PA, Physical activity

SB, Sedentary behaviors
