# Supplementary material for: Movement behavior policies in the early childhood education and care setting: An international scoping review
Source: Front Public Health. 2023 Apr 11;11:1077977. doi: 10.3389/fpubh.2023.1077977 (PMC10126357; doi:10.3389/fpubh.2023.1077977)
Supplement: Supplementary file 4 [file Table_4.DOCX]

**Supp. Table 1. ECEC time-specific physical activity recommendations 2010-2021.**

|  | **Year** | **Jurisdiction** | **Age group** | **Physical Activity (PA) (mins/day)** | | | | |
| --- | --- | --- | --- | --- | --- | --- | --- | --- |
| **Source** |  |  |  | **Total PA** | **Vigorous PA** | **Moderate -Vigorous PA** | **Light PA** | **Tummy Time** |
| National Center on Early Childhood Quality Assurance (53) | 2021 | USA |  |  |  |  |  |  |
|  | 2018 | Alaska | 0-6yrs | 60 | - | 30 | - | - |
|  | 2020 | Arkansas |  | 60 | - | - | - | 30 |
|  | 2017 | Colorado |  | - | - | 60 | - | - |
|  | 2015 | Connecticut |  | - | - | - | - | 30 (2 x daily) |
|  | 2020 | Delaware |  | 60 | - | - | - | daily |
|  | 2019 | Florida |  | - | - | - | - | - |
|  | 2013 | Indiana |  | 120 | - | - | - | - |
|  | 2015 | Iowa |  | - | - | - | - | - |
|  | 2020 | Kansas |  | 60 | - | - | - | - |
|  | 2018 | Kentucky |  | 60 | - | - | - | - |
|  | 2019 | Louisiana |  | - | - | - | - | - |
|  | 2020 | Maryland |  | - | - | - | - | daily |
|  | 2010 | Massachusetts |  | - | - | - | - | - |
|  | 2020 | Michigan |  | - | - | 60 | 15 | - |
|  | 2019 | Minnesota |  | - | - | - | - | - |
|  | 2020 | Mississippi |  | - | - | - | - | - |
|  | 2018 | Montana |  | - | - | - | - | - |
|  | 2013 | Nebraska |  | 60 | - | - | - | - |
|  | 2017 | Nevada |  | 30 (care <4hrs)  60 (care ≥4hrs) | - | - | - | 2 x daily |
|  | 2017 | New Hampshire |  | 60 | - | - | - | - |
|  | 2017 | New Jersey |  | - | - | - | - | - |
|  | 2016 | New Mexico |  | <2yrs: 30  ≥2yrs: 60 | - | - | - | - |
|  | 2020 | North Carolina |  | - | - | - | - | - |
|  | 2016 | North Dakota | 0-6yrs | - | - | - | - | - |
|  | 2020 | Ohio |  | - | - | - | - | - |
|  | 2020 | Oklahoma |  | - | - | - | - | - |
|  | 2019 | Oregon |  | 60 | - | - | - | - |
|  | 2013 | South Dakota |  | - | 20 | 2<3yrs: 60-90  3-5yrs: 90-120 | - | 3-5 x multiple |
|  | 2019 | Tennessee |  | 60 | - | - | - | - |
|  | 2021 | Texas |  | 30 (care <4hrs)  60 (care ≥4hrs) | - | - | - | - |
|  | 2020 | Utah |  | 60-90 | <3yrs: 60  ≥3yrs: 90 | - | - | - |
|  | 2020 | Vermont |  | 60-90 | <2yrs: 20/3hrs ≥2yrs: 30/ 3hrs | - | - | - |
|  | 2020 | Washington |  | 30 | - | - | - | - |
|  | 2016 | Wisconsin |  | 180 | - | - | - | - |
|  | 2016 | Wyoming |  | - | - | - | - | daily |
| Arizona Department of Health Services, Arizona Nutrition Network (69) | 2016 | Arizona, USA | 0-6yrs | - | - | ≥1yr: 60 | - | 3-5 x multiple |
| Benjamin-Neelon S, et al (97) | 2018 | USA | Infants | - | - | - | - | daily |
| Department of Education and Early Childhood Development (33) | 2017 | Newfoundland & Labrador, Canada | 0-5yrs | 60 AM  60 PM | - | - | - | - |
| Broyles S (61) | 2013 | Louisiana, USA | 1-5yrs | 60 | - | - | - | - |
| The P.E.I. Healthy Eating Alliance (73) | 2016 | Prince Edward Island, Canada | 0-5yrs | ≥18mth: 60 | - | - | - | - |
| Buran M & Parham-Lee M (36) | 2018 | California, USA | 0-5yrs | 3-5yrs: 120 | - | - | - | 3-5 x multiple |
| ChangeLab Solutions (66) | 2013 | USA | 0-6yrs | - | - | 1<3yrs: 60-120  3-6yrs: 90-120 | - | - |
| Australian Government Department of Health and Ageing (43) | 2013 | Australia | 0-5yrs | 180 | - | - | - | - |
| Province of British Columbia (72) | 2016 | British Columbia, Canada | 0-5yrs | 120 | - | 60 | - | daily |
| Ministry of Education and Culture (77) | 2016 | Finland | 0-8yrs | 180 | 60 | - | - | - |
| New Zealand Ministry of Health (12) | 2017 | New Zealand | 0-5yrs | ≥2yrs: 180 | - | - | - | - |
| Play Scotland (55) | 2021 | Scotland, UK | 0-4yrs | ≥1yr: 180 | - | 3-4yrs: 60 | - | 30 |
| State of Alaska Department of Health and Social Services (49) | 2020 | Alaska, USA | 0-5yrs | 3-5yrs: 60 | >18mth: 30 | - | - | daily |
| Startsmart@school (48) | 2020 | Hong Kong | 2-6yrs | 2<3yrs: 180  3-6yrs: 180 | - | 3-6yrs: 60 | - | - |
| Pennsylvania Chapter of the American Academy of Pediatrics (66) | 2014 | USA | 0-6yrs | - | - | 1<3yrs: 60-90  3-5yrs: 90-120 | - | 3-5 x multiple |
| NSW Health (47) | 2020 | New South Wales, Australia | 0-5yrs | 1<3yrs: 180  3-5yrs: 180 | 3-5yrs: 60 | - | - | 30 |
| North Dakota Department of Health, North Dakota, et al (59) | 2016 | North Dakota, USA | 0-5yrs | - | - | 1<3yrs: 60-90  3-5yrs: 90-120 | - | daily |
| New York City Health. (67) | 2019 | New York, USA | 0-12yrs | ≥1yr: 60 | - | - | - | - |
| Missouri Department of Health and Senior Services (40) | 2018 | Missouri, USA | 0-6yrs | - | - | - | - | 3-5 x multiple |
| Ministry of Education Ontario (42) | 2019 | Ontario, Canada | 0-5yrs | - | - | - | - | - |
| Hughes D (58) | 2013 | USA | 0-18yrs | 1<3yrs: 60-90  3-5yrs: 120 | - | - | - | 3-5 x multiple |
| New York City Health. (62) | 2011 | New York, USA | 0-5yrs | ≥1yr: 60 | - | - | - | - |
| Maine Health (64) | 2015 | Maine, USA | 0-5yrs | 60 | - | - | - | - |
| Harvard TH Chan School of Public Health (60) | 2011 | Massachusetts, USA | 0-5yrs | 2<3yrs: 60-90  3-5yrs: 90-120 | - | - | - | daily |
| Goodfellow A, et al (38) | 2018 | Scotland, UK | 0-16yrs | ≥18m: 180 | - | - | - | - |
| The Nemours Foundation (56) | 2021 | USA | 0-5yrs | - | - | 1<2yrs: 60  2-5yrs: 120 | - | 3-5 x multiple |
| Evans T, et al (57) | 2011 | Wisconsin, USA | 0-5yrs | 2<3yrs: 60-90  3-5yrs: 90-120 | - | - | - | - |
| Early Childhood Iowa State Board, et al (37) | 2018 | Iowa, USA | 0-5yrs | - | - | - | - | 2 x daily |
| Colorado Office of Early Childhood (45) | 2010 | Colorado, USA | 0-5yrs | - | ≥3yrs: daily | - | - | daily |
| DeCourcey M (74) | 2016 | New Brunswick, Canada | 0-5yrs | 180 | - | 60 | - | - |
| British Heart Foundation (76) | 2012 | UK | 0-5yrs | ≥18mth: 180 | - | - | - | - |
| Tabak RG, et al (68) | 2013 | Mississippi, USA | 0-1yrs | 30 | - | - | - | - |
| Piercy KL, et al (41) | 2018 | USA | 0+yrs | 180 | - | - | - | - |
| Draper CE, et al (50) | 2021 | South Africa | 0-5yrs | 1<3yrs: 180  3-5yrs: 180 | - | - | - | 30 |
| Christian, HE, et al (44) | 2020 | Perth, Australia | 0-5yrs | ≥1yr: 120-150 | - | - | - | 30 |
| Government of Nova Scotia (51) | 2021 | Nova Scotia, Canada | 0-5yrs | - | - | - | - | - |
| Ministry of Education Quebec (52) | 2021 | Quebec, Canada | 0-5yrs | 60 | - | - | - | - |
| National Health and Safety Performance Standards (54) | 2021 | USA | 0-6yrs | - | - | 1<3yrs: 60-90  3-5yrs: 90-120 | - | 3-5 x multiple |
| Institute of Medicine (24) | 2011 | USA | 0-5yrs | 15/hr | - | - | - | - |
| Weisman, J (70) | 2014 | USA | 0-5yrs | - | 1<3yrs: 60-90  3-5yrs: 90-120 | - | - | daily |
| Healthy Child Manitoba (71) | 2014 | Manitoba, Canada | 0-12yrs | - | - | - | - | daily |
| Department of Education Government of Nunavut (75) | 2014 | Nunavut, Canada | 0-5yrs | 30 | - | - | - | - |
