# Supplementary material for: Movement behavior policies in the early childhood education and care setting: An international scoping review
Source: Front Public Health. 2023 Apr 11;11:1077977. doi: 10.3389/fpubh.2023.1077977 (PMC10126357; doi:10.3389/fpubh.2023.1077977)
Supplement: Supplementary file 5 [file Table_5.DOCX]

**Supp. Table 2. ECEC time-specific other recommendations 2010-2021.**

| **Source** | **Year** | **Jurisdiction** | **Age group** | **Other physical activity (PA) types** | | | | **Sedentary Behavior** | **Screen Time^a^** | |  |
| --- | --- | --- | --- | --- | --- | --- | --- | --- | --- | --- | --- |
|  |  |  |  | **Structured PA** | **Unstructured PA** | **Outside PA** | **Inside PA** |  | **TV/Screen overall** | **Computer** | **Sleep (hrs/day)** |
| National Center on Early Childhood Quality Assurance (53) | 2021 | USA | 0-6yrs |  |  |  |  |  |  |  |  |
|  | 2018 | Alaska |  | - | - | <1yr: 3 x week  ≥1yr: daily | - | - | <2yrs: 0  ≥2yrs: 60 | ≥2yrs: 120 | - |
|  | 2020 | Arkansas |  | - | - | daily | Match to TPA | - | <2yrs: 0  ≥2yrs: 30 | ≥2yrs:30 | 30 (care ≥5hrs) |
|  | 2017 | Colorado |  | - | - | daily | - | 30 | <2yrs: 0  ≥2yrs: 60 | - | 2-3yrs: 30 |
|  | 2015 | Connecticut |  | - | - | 2 x daily | - | - | <2yrs: 0  ≥2yrs  60-120 | - | - |
|  | 2020 | Delaware |  | - | - | daily | - | - | 120 | - | - |
|  | 2019 | Florida |  | - | - | daily | - | - | - | - | - |
|  | 2013 | Indiana |  | - | - | daily | - | 30 | - | - | - |
|  | 2015 | Iowa |  | - | - | - | - | - | 120 | - | <120  (care ≥4hrs) |
|  | 2020 | Kansas |  | - | - | - | - | 30 | <2yrs: 0  ≥2yrs: 60 | - | < 4yrs: 75  (care ≥5hrs) |
|  | 2018 | Kentucky |  | - | - | - | - | - | - | - | 60 (care ≥4hrs)  10 (care <4hrs) |
|  | 2019 | Louisiana |  | - | - | 2 x daily | - | - | 60 | - | - |
|  | 2020 | Maryland |  | - | - | - | - | 30 | <2yrs: 0  >2yrs: 25 | - | - |
|  | 2010 | Massachusetts | 0-6yrs | -  -  -  -  -  -  -  -  -  -  -  -  -  -  -  - | - | - | - | 30 | - | - | - |
|  | 2020 | Michigan |  |  | - | - | - | 30 | <2yrs: 0  ≥2yrs: 60 | - | 60-90  (care ≥6hrs) |
|  | 2019 | Minnesota |  |  | - | - | - | 30 | - | - | 30 |
|  | 2020 | Mississippi |  |  | - | - | - | 30 | - | - | - |
|  | 2018 | Montana |  |  | - | ≥1yr: daily | - | 15 | - | - | - |
|  | 2013 | Nebraska |  |  | - | - | - | 15/2hrs | - | - | 60 |
|  | 2017 | Nevada |  |  | - | - | - | 30 | <2yrs: 0 | - | - |
|  | 2017 | New Hampshire |  |  | - | daily | - | - | <2yrs: 0  ≥2yrs: 20 | - | - |
|  | 2017 | New Jersey |  |  | - | daily | - | 15 | <2yrs: 0 | <2yrs: 0 | - |
|  | 2016 | New Mexico |  |  | - | daily | - | - | <3yrs: 0  ≥3yrs: 30 | - | - |
|  | 2020 | North Carolina |  |  | - | ≥1yr: daily | - | 30 | - | - | - |
|  | 2016 | North Dakota |  |  | - | - | - | 20 | <1yr: 0  ≥1yr: 30 | - | - |
|  | 2020 | Ohio |  |  | - | daily | - | - | - | - | - |
|  | 2020 | Oklahoma |  |  | - | daily | - | - | - | - | - |
|  | 2019 | Oregon |  |  | - | - | - | 15 | <18mth: 0  ≥18mth: 30 | - | - |
|  | 2013 | South Dakota |  |  | - | 60 + 2 x daily | - | 15 | <2yrs: 0  ≥2yrs: 60 | - | - |
|  | 2019 | Tennessee |  | - | - | daily | - | 30 | <18mth: 0  ≥18mth: 60 | - | <120 |
|  | 2021 | Texas | 0-6yrs | - | - | daily | - | - | <2yrs: 0  ≥2yrs: 30 | - | - |
|  | 2020 | Utah |  | - | - | ≥1yr: 2-3 x daily | Match to VPA | 15 | <2yrs: 0  ≥2yrs: 15 | - | ≥2yrs: 60  (care ≥4hrs) |
|  | 2020 | Vermont |  | - | - | - | - | 15 | <2yrs: 0  ≥2yrs: 30 | - | - |
|  | 2020 | Washington |  | - | - | daily | - | - | - | - | - |
|  | 2016 | Wisconsin |  | - | - | - | - | 60 | <2yrs: 0 | <2yrs: 0 | - |
|  | 2016 | Wyoming |  | - | - |  | - | - | - | - | - |
| Arizona Department of Health Services, Arizona Nutrition Network (69) | 2016 | Arizona, USA | 0-6yrs | 1-6yrs: 30 | 1-6yrs: 30 | - | - | 60 | <2yrs: 0  ≥2yrs: 30-40 | - | - |
| Benjamin-Neelon S, et al (97) | 2018 | USA | Infants | - | - | - | - | - | - | - | - |
| Department of Education and Early Childhood Development (33) | 2017 | Newfoundland & Labrador, Canada | 0-5yrs | - | - | 45 AM  45 PM | - | - | <2yrs 0 | - | daily |
| Broyles S (61) | 2013 | Louisiana, USA | 1-5yrs | 1<3yrs: 30  3-5yrs: 60 | - | - | - | - | <2yrs: 0  ≥2yrs: 30 | - | - |
| The P.E.I. Healthy Eating Alliance (73) | 2016 | Prince Edward Island, Canada | 0-yrs |  | daily | daily | - | - | <2yrs: 0  ≥2yrs: 30 | - | - |
| Buran M & Parham-Lee M (36) | 2018 | California, USA | 0-5yrs | 1<3yrs: 30  3-5yrs: 60 + 2 x daily | 1-3yrs: 60  3-5yrs: 60 | <1yr: 2-3 x daily  >1yr: 60-90 +  2 x daily | - | <1yr: 15  1-3yrs: 60 | <1yr: 0 | - | 3-5yrs: 120 |
| ChangeLab Solutions (66) | 2013 | USA | 0-6yrs | ≥1yr: 2 x daily | - | <1yr: daily  ≥1yr: 2 x daily | - | <1yr: 15  ≥1yr: 60 | <2yrs: 0  ≥2yrs: 30-60 | <2yrs: 0  ≥2yrs: 30-60 | - |
| Australian Government Department of Health and Ageing (43) | 2013 | Australia | 0-5yrs | - | - | - | - | 60 | <2yrs: 0  ≥2yrs: 60 | <2yrs: 0  ≥2yrs: 60 | - |
| Province of British Columbia (72) | 2016 | British Columbia, Canada | 0-5yrs | daily | daily |  |  |  |  |  |  |
| Ministry of Education and Culture (77) | 2016 | Finland | 0-8yrs | - | - | 120 | - | - | - | - | - |
| New Zealand Ministry of Health (12) | 2017 | New Zealand | 0-5yrs | - | - | - | - | - | <2yrs: 0  ≥2yrs: 60 | - | - |
| Play Scotland (55) | 2021 | Scotland, UK | 0-4yrs | - | - | - | - | - | - | - | - |
| State of Alaska Dept of Health and Social Services (49) | 2020 | Alaska, USA | 0-5yrs | - | <18mth: daily  ≥18mth: 60 | ≥18mth: daily | - | - | <18mth: 0  ≥18mth: 20 | - | - |
| Startsmart@school (48) | 2020 | Hong Kong | 2-6yrs | - | - | - | - | 60 | 60 | - | - |
| Pennsylvania Chapter of the American Academy of Pediatrics (66) | 2014 | USA | 0-6yrs | 2 x daily | - | <1yr: 2-3 x daily  ≥1yr: 60-90 | Match to outdoor | <1yr: 15 | <2yrs: 0  ≥2yrs: 30 | ≥2yrs:15 | - |
| NSW Health (47) | 2020 | New South Wales, Australia | 0-5yrs | - | - | - | - | 60 | <2yrs: 0  ≥2yrs:60 | - | - |
| North Dakota Department of Health, North Dakota, et al (59) | 2016 | North Dakota, USA | 0-5yrs | 1<3yrs: 30  3-5yrs: 60 | 1<3yrs: 30  3-5yrs: 60 | 60-90 + 2x daily | - | 15 | <2yrs: 0  ≥2yrs: 30 | - | - |
| New York City Health (67) | 2019 | New York, USA | 0-12yrs | ≥1yr: 30 | - | - | - | 30 | <2yrs: 0  ≥2yrs: 15 (max. 30/wk) | - | - |
| Missouri Department of Health and Senior Services (40) | 2018 | Missouri, USA | 0-6yrs | ≥1yr: 30 | ≥1yr: 60 | <1yr: 2-3 x daily | - | <1yr: 15 | <2yrs: 0  ≥2yrs: 30 | - | - |
| Ministry of Education Ontario (42) | 2019 | Ontario, Canada | 0-5yrs | - | - | 120 | - | - | - | - | <120 |
| Hughes D (58) | 2013 | USA | 0-18yrs | daily | daily | <2yr: 2-3 x daily  ≥2yrs: 60-90 | - | <2yrs: 15  ≥2yrs: 60 | <2yrs: 0  ≥2yrs: 30/wk | >2yrs: 15 | - |
| New York City Health (62) | 2011 | New York, USA | 0-5yrs | ≥3yr: 30 | - | daily | - | 60 | <2yrs: 0  ≥2yrs:60 | - | - |
| Maine Health (64) | 2015 | Maine, USA | 0-5yrs | - | - | - | - | - | 120 | - | - |
| Harvard TH Chan School of Public Health (60) | 2011 | Massachusetts, USA | 0-5yrs | 2 x daily | - | 2-3 x daily | - | 15-30 | <2yrs: 0  ≥2yrs: 30/week | - | - |
| Goodfellow A, et al (38) | 2018 | Scotland, UK | 0-16yrs | daily | daily | daily | - | - | - | - | - |
| The Nemours Foundation (56) | 2021 | USA | 0-5yrs | ≥1yr: daily | ≥1yr: daily | ≥1yr: daily | - | - | <2yr: 0  ≤2yrs: 60 | - | - |
| Evans T, et al (57) | 2011 | Wisconsin, USA | 0-5yrs | <1yr: daily  2<3yrs: 30  3-5yrs: 60 | <1yr: daily  2<3yrs: 30  3-5yrs: 60 | <1yr: daily  >2yrs: 2-3 x daily | - | ≥2yrs: 15 (max 60) | - | - | - |
| Early Childhood Iowa State Board, et al (37) | 2018 | Iowa, USA | 0-5yrs | 3-5yrs: daily | 3-5yrs: daily | daily | - | <1yr: 15 | - | - | - |
| Colorado Office of Early Childhood (45) | 2010 | Colorado, USA | 0-5yrs | - | <18mth: daily | ≥18mth: daily | - | - | - | - | - |
| DeCourcey M. (74) | 2016 | New Brunswick, Canada | 0-5yrs | - | Daily | 160 | - | - | 0 | - | - |
| British Heart Foundation (76) | 2012 | UK | 0-5yrs | - | - | - | - | - | - | - | - |
| Tabak RG, et al (68) | 2013 | Mississippi, USA | 0-18yrs | - | - | - | - | - | - | - | - |
| Piercy KL, et al (41) | 2018 | USA | 0+yrs | - | - | - | - | - | - | - | - |
| Draper CE, et al (50) | 2021 | South Africa | 0-5yrs | - | - | - | - | 60 | <2yr: 0  ≥2yrs: 60 | - | - |
| Christian, HE, et al (44) | 2020 | Perth, Australia | 0-5yrs | - | - | - | - | 60 | 0 | - | - |
| Government of Nova Scotia (51) | 2021 | Nova Scotia, Canada | 0-5yrs | - | 45 AM  45 PM | 30 | - | - | - | - | - |
| Ministry of Education Quebec (52) | 2021 | Quebec, Canada | 0-5yrs | - | - | - | - | - | - | - | - |
| National Health and Safety Performance Standards (54) | 2021 | USA | 0-6yrs | 2 x daily | 2 x daily | <1yr: 2-3 x daily  ≥1yr: 60-90 | Match to outdoor | <1yr: 15 | <2yr: 0  ≥2yrs: 60 | - | - |
| Institute of Medicine (24) | 2011 | USA | 0-5yrs | - | - | - | - | - | 2-5yrs: 120 | - | - |
| Weisman, J (70) | 2014 | USA | 0-5yrs | - | - | <1yr: 2-3 x daily  ≥1yr: 60-90 | - | - | <2yr: 0  ≥2yrs: 30/week | - | - |
| Healthy Child Manitoba (71) | 2014 | Manitoba, Canada | 0-12yrs | - | daily | daily | - | 30 | - | - | daily |
| Department of Education Government of Nunavut (75) | 2014 | Nunavut, Canada | 0-5yrs | - | - | daily | - | - | - | - | daily |
