# Supplementary material for: Movement behavior policies in the early childhood education and care setting: An international scoping review
Source: Front Public Health. 2023 Apr 11;11:1077977. doi: 10.3389/fpubh.2023.1077977 (PMC10126357; doi:10.3389/fpubh.2023.1077977)
Supplement: Supplementary file 6 [file Table_6.DOCX]

**Supp. Table 3. ECEC movement behavior policies overview, 2010-2021.**

| **Category** | **Sub-category** | **Source** |
| --- | --- | --- |
| Publication year | 2017-mid 2021 | (12, 33, 34, 36-45, 47-56, 97, 98) |
|  | 2010-2016 | (57-62, 64-75, 77, 99, 100) |
| Policy jurisdiction | National | (12, 39, 41, 43, 48, 50, 53, 54, 56, 58, 65, 66, 70, 77, 97, 99, 100) |
|  | Subnational | (33, 34, 36-38, 40, 42, 44, 45, 47, 51, 52, 55, 57, 59-62, 64, 67-69, 71-75, 98) |
| Country of origin | USA | (34, 36, 37, 40, 41, 45, 49, 53, 54, 56-62, 64-69, 97, 100) |
|  | Canada | (33, 39, 42, 51, 52, 71-75) |
|  | Australia | (43, 44, 47) |
|  | UK | (38, 55, 99) |
|  | Finland | (77) |
|  | South Africa | (50) |
|  | Hong Kong | (48) |
|  | New Zealand | (12) |
| Document types | Website/Online database | (12, 33, 36-40, 42, 43, 45, 47-49, 51-60, 62, 64, 65, 67, 69-75, 77, 99) |
|  | Journal articles | (34, 41, 44, 50, 68, 97, 100) |
|  | Policy briefs/legislation | (61, 66, 98) |
| Age groupings | 0-5 years | (12, 33, 36, 37, 42-45, 47, 49-52, 55-57, 59-62, 64, 72-75, 97, 99) |
|  | 0-5 years and <18 years | (38, 40, 48, 53, 54, 58, 65-69, 71, 77) |
| Policy sector | Health | (12, 33, 34, 36, 38-41, 43-45, 47-50, 54, 56-62, 64-70, 72-74, 77, 97, 99, 100) |
|  | Education | (33, 34, 37, 39, 41, 42, 44, 45, 47-54, 57, 59-61, 65, 69-75, 77, 98, 99) |
|  | Sport & recreation | (12, 39, 41, 44, 48, 50, 52, 55, 70, 73, 74) |
|  | Environment | (34, 36, 39, 41, 55, 64) |
|  | Rural & urban planning | (39, 41, 55, 67) |
|  | Research | (24, 41, 44, 47, 60, 63, 64, 66) |
|  | Transport | (39, 41, 70) |
| Policy focus | Upstream | (34, 37, 39, 41, 44, 50-53, 55, 61, 66, 68, 70, 97, 98) |
|  | Downstream | (12, 33, 36, 38, 40, 42, 43, 45, 47-49, 53, 54, 56-60, 62, 64, 65, 67, 69, 71-75, 77, 99, 100) |
| Policy type | Legislated | (33, 37, 42, 49, 51, 53, 67, 71, 72, 75, 77, 97, 98) |
|  | Not legislated | (12, 34, 36, 38-41, 43-45, 47, 48, 50, 52, 54-62, 64-66, 68-70, 73-75, 99, 100) |
| Policy cycle stage | Agenda setting | (41, 68, 70) |
|  | Formulation | (39, 44, 60, 61, 100) |
|  | Implementation | (12, 33, 36-38, 40, 42, 45, 48, 49, 51, 52, 56-58, 60, 62, 64, 66, 67, 69, 72, 74, 77, 98, 99) |
|  | Evaluation | (34, 50, 97) |
|  | Maintenance | (43, 47, 53-55, 65, 73, 75) |
| Policy themes | Address rising obesity, increased sedentary behaviors, decreased physical activity | (12, 36, 47, 50, 56, 58, 61, 64, 68, 69, 72, 100) |
|  | Standardize and clarify movement behaviors while attending ECEC | (37, 40, 43-45, 49, 51, 53, 62, 65-67, 71, 75, 77, 97, 98, 100) |
|  | Help children reach their full potential through optimal health and development | (33, 34, 41, 42, 50, 52, 54, 55, 57, 74, 77) |
|  | Address overall health inequities and reduce risk of short- and long-term chronic disease | (33, 34, 38, 39, 41, 48, 50, 52, 60, 64, 74, 99) |
|  | Build capacity to support healthy movement behaviors and community environments for physical activity | (34, 40, 53-55, 57, 58, 64, 68, 70, 71, 73, 77, 99) |
| Policy stakeholders | Government departments | (12, 33, 34, 36, 37, 39-41, 43-45, 47-54, 56, 57, 59, 60, 62, 66, 67, 69-75, 77, 97-100) |
|  | Non-government organizations | (36, 37, 39, 41, 43-45, 47-50, 52, 54-60, 64, 65, 67, 69-71, 73, 74, 77) |
|  | Working/reference group | (12, 37, 39, 41, 43-45, 47, 48, 50, 54, 57, 58, 60, 64, 67, 70, 77, 99) |
|  | University/academics | (34, 36, 37, 39, 41, 43-45, 47-50, 54, 55, 57-61, 65-68, 77, 97, 99, 100) |
|  | User end-group (ECEC staff) | (34, 37, 39, 41-45, 47-50, 54, 55, 57, 58, 60, 65, 67-69, 71, 73-75, 77, 97, 100) |
| Main stakeholder guiding values | Life course perspective of the health and well-being of young children | (12, 37, 40, 43, 45, 48, 50, 56, 57, 61, 66, 67, 97, 99) |
|  | Rights of children to learn, grow and develop in safe and healthy environments | (38, 47, 48, 50, 53-55, 59, 60, 77) |
|  | Integration of families, educators and communities to support healthy habits | (36, 43, 47, 49, 56-58, 60, 64, 67, 69) |
|  | Physical activity in ECEC through best practice policy | (34, 41, 44, 58, 59, 62, 65, 68, 70, 100) |
| Political will | Present | (34, 37, 39-41, 43, 44, 47, 52, 55, 56, 61, 64-66, 70, 73, 77) |
|  | Not present | (12, 33, 36, 38, 42, 43, 45, 48-51, 53, 54, 57-60, 62, 67-69, 71, 72, 74, 75, 97, 99, 100) |
| Implementation supports | Resource/practice guide; training | (12, 33, 36-38, 40-45, 47-51, 54-60, 62, 64-69, 71-75, 77, 97, 99) |
|  | Dedicated website | (12, 36-40, 43, 44, 47-50, 54-56, 58, 60, 64, 68, 69, 71, 73-75, 77, 99, 100) |
|  | Implementation tools | (33, 36, 38-40, 42, 44, 45, 47, 48, 50, 52, 55-58, 60, 64, 66, 67, 69, 71, 73, 75, 77, 99) |
|  | Self-evaluation tools | (36, 38, 40, 44, 47, 48, 55-58, 62, 64, 67, 69) |

| **Category** | **Sub-category** | **Source** |
| --- | --- | --- |
| Publication year | 2017-mid 2021 | (12, 32, 33, 35-44, 46-55, 96, 97) |
|  | 2010-2016 | (56-61, 63-74, 76, 98, 99) |
| Policy jurisdiction | National | (12, 38, 40, 42, 47, 49, 52, 53, 55, 57, 64, 65, 69, 76, 96, 98, 99) |
|  | Subnational | (32, 33, 35-37, 39, 41, 43, 44, 46, 50, 51, 54, 56, 58-61, 63, 66-68, 70-74, 97) |
| Country of origin | USA | (33, 35, 36, 39, 40, 44, 48, 52, 53, 55-61, 63-68, 96, 99) |
|  | Canada | (32, 38, 41, 50, 51, 70-74) |
|  | Australia | (42, 43, 46) |
|  | UK | (37, 54, 98) |
|  | Finland | (76) |
|  | South Africa | (49) |
|  | Hong Kong | (47) |
|  | New Zealand | (12) |
| Document types | Website/Online database | (12, 32, 35-39, 41, 42, 44, 46-48, 50-59, 61, 63, 64, 66, 68-74, 76, 98) |
|  | Journal articles | (33, 40, 43, 49, 67, 96, 99) |
|  | Policy briefs/legislation | (60, 65, 97) |
| Age groupings | 0-5 years | (12, 32, 35, 36, 41-44, 46, 48-51, 54-56, 58-61, 63, 71-74, 96, 98) |
|  | 0-5 years and <18 years | (37, 39, 47, 52, 53, 57, 64-68, 70, 76) |
| Policy sector | Health | (12, 32, 33, 35, 37-40, 42-44, 46-49, 53, 55-61, 63-69, 71-73, 76, 96, 98, 99) |
|  | Education | (32, 33, 36, 38, 40, 41, 43, 44, 46-53, 56, 58-60, 64, 68-74, 76, 97, 98) |
|  | Sport & recreation | (12, 38, 40, 43, 47, 49, 51, 54, 69, 72, 73) |
|  | Environment | (33, 35, 38, 40, 54, 63) |
|  | Research | (40, 43, 46, 59, 62, 63, 65) |
|  | Rural & urban planning | (38, 40, 54, 66) |
|  | Transport | (38, 40, 69) |
| Policy focus | Upstream | (33, 36, 38, 40, 43, 49-52, 54, 60, 65, 67, 69, 96, 97) |
|  | Downstream | (12, 32, 35, 37, 39, 41, 42, 44, 46-48, 52, 53, 55-59, 61, 63, 64, 66, 68, 70-74, 76, 98, 99) |
| Policy type | Legislated | (32, 36, 41, 48, 50, 52, 66, 70, 71, 74, 76, 96, 97) |
|  | Not legislated | (12, 33, 35, 37-40, 42-44, 46, 47, 49, 51, 53-61, 63-65, 67-69, 72-74, 98, 99) |
| Policy cycle stage | Agenda setting | (40, 67, 69) |
|  | Formulation | (38, 43, 59, 60, 99) |
|  | Implementation | (12, 32, 35-37, 39, 41, 44, 47, 48, 50, 51, 55-57, 59, 61, 63, 65, 66, 68, 71, 73, 76, 97, 98) |
|  | Evaluation | (33, 49, 96) |
|  | Maintenance | (42, 46, 52-54, 64, 72, 74) |
| Policy themes | Address rising obesity, increased sedentary behaviors, decreased physical activity | (12, 35, 46, 49, 55, 57, 60, 63, 67, 68, 71, 99) |
|  | Standardize and clarify movement behaviors while attending ECEC | (36, 39, 42-44, 48, 50, 52, 61, 64-66, 70, 74, 76, 96, 97, 99) |
|  | Help children reach their full potential through optimal health and development | (32, 33, 40, 41, 49, 51, 53, 54, 56, 73, 76) |
|  | Address overall health inequities and reduce risk of short- and long-term chronic disease | (32, 33, 37, 38, 40, 47, 49, 51, 59, 63, 73, 98) |
|  | Build capacity to support healthy movement behaviors and community environments for physical activity | (33, 39, 52-54, 56, 57, 63, 67, 69, 70, 72, 76, 98) |
| Policy stakeholders | Government departments | (12, 32, 33, 35, 36, 38-40, 42-44, 46-53, 55, 56, 58, 59, 61, 65, 66, 68-74, 76, 96-99) |
|  | Non-government organizations | (35, 36, 38, 40, 42-44, 46-49, 51, 53-59, 63, 64, 66, 68-70, 72, 73, 76) |
|  | Working/reference group | (12, 36, 38, 40, 42-44, 46, 47, 49, 53, 56, 57, 59, 63, 66, 69, 76, 98) |
|  | University/academics | (33, 35, 36, 38, 40, 42-44, 46-49, 53, 54, 56-60, 64-67, 76, 96, 98, 99) |
|  | User end-group (ECEC staff) | (33, 36, 38, 40-44, 46-49, 53, 54, 56, 57, 59, 64, 66-68, 70, 72-74, 76, 96, 99) |
| Main stakeholder guiding values | Life course perspective of the health and well-being of young children | (12, 36, 39, 42, 44, 47, 49, 55, 56, 60, 65, 66, 96, 98) |
|  | Rights of children to learn, grow and develop in safe and healthy environments | (37, 46, 47, 49, 52-54, 58, 59, 76) |
|  | Integration of families, educators and communities to support healthy habits | (35, 42, 46, 48, 55-57, 59, 63, 66, 68) |
|  | Physical activity in ECEC through best practice policy | (33, 40, 43, 57, 58, 61, 64, 67, 69, 99) |
| Political will | Present | (33, 36, 38-40, 42, 43, 46, 51, 54, 55, 60, 63-65, 69, 72, 76) |
|  | Not present | (12, 32, 35, 37, 41, 42, 44, 47-50, 52, 53, 56-59, 61, 66-68, 70, 71, 73, 74, 96, 98, 99) |
| Implementation supports | Resource/practice guide; training | (12, 32, 35-37, 39-44, 46-50, 53-59, 61, 63-68, 70-74, 76, 96, 98) |
|  | Dedicated website | (12, 35-39, 42, 43, 46-49, 53-55, 57, 59, 63, 67, 68, 70, 72-74, 76, 98, 99) |
|  | Implementation tools | (32, 35, 37-39, 41, 43, 44, 46, 47, 49, 51, 54-57, 59, 63, 65, 66, 68, 70, 72, 74, 76, 98) |
|  | Self-evaluation tools | (35, 37, 39, 43, 46, 47, 54-57, 61, 63, 66, 68) |
